# Vitamin D and Its Potential Interplay With Pain Signaling Pathways

**DOI:** 10.3389/fimmu.2020.00820

**Published:** 2020-05-28

**Authors:** Abdella M. Habib, Karim Nagi, Nagendra Babu Thillaiappan, VijayaKumar Sukumaran, Saghir Akhtar

**Affiliations:** College of Medicine, QU Health, Qatar University, Doha, Qatar

**Keywords:** nociception, DRG, VDR, NGF, EGFR, GDNF, opioids, vitamin D toxicity

## Abstract

About 50 million of the U.S. adult population suffer from chronic pain. It is a complex disease in its own right for which currently available analgesics have been deemed woefully inadequate since ~20% of the sufferers derive no benefit. Vitamin D, known for its role in calcium homeostasis and bone metabolism, is thought to be of clinical benefit in treating chronic pain without the side-effects of currently available analgesics. A strong correlation between hypovitaminosis D and incidence of bone pain is known. However, the potential underlying mechanisms by which vitamin D might exert its analgesic effects are poorly understood. In this review, we discuss pathways involved in pain sensing and processing primarily at the level of dorsal root ganglion (DRG) neurons and the potential interplay between vitamin D, its receptor (VDR) and known specific pain signaling pathways including nerve growth factor (NGF), glial-derived neurotrophic factor (GDNF), epidermal growth factor receptor (EGFR), and opioid receptors. We also discuss how vitamin D/VDR might influence immune cells and pain sensitization as well as review the increasingly important topic of vitamin D toxicity. Further *in vitro* and *in vivo* experimental studies will be required to study these potential interactions specifically in pain models. Such studies could highlight the potential usefulness of vitamin D either alone or in combination with existing analgesics to better treat chronic pain.

## Introduction

The newly proposed definition of pain by the International Association for the Study of Pain states—“An aversive sensory and emotional experience typically caused by, or resembling that caused by, actual or potential tissue injury^1^”. Pain represents the body's alarm system and serves as an alert to danger. Chronic pain is a complex disease in its own right for which currently available analgesics have been deemed woefully inadequate since ~20% of the sufferers derive no benefit. This is thought to be due to the complexity and plasticity of chronic pain, and the underpinning mechanisms are not clear as yet. About 50 million of the U.S. adult population suffer from chronic pain, and regardless of the reason, the current treatments primarily manage the condition rather than provide a cure (1).

Vitamin D, commonly identified as a fat-soluble vitamin, is known for its role in calcium homeostasis and bone metabolism. Recent studies have linked vitamin D status and its receptor activity with several health conditions, including development of chronic pain,^1^ the leading cause of disability and disease burden globally (2). Studies on the association between vitamin D status and incidence of chronic pain have been contradictory (3, 4). There are potentially many reasons for the variations in the clinical trial studies. However, a recent well-controlled study in Europeans has shown that reduced vitamin D levels were significantly associated with painful diabetic peripheral neuropathy (5). A strong correlation is also shown for hypovitaminosis D and bone pain (6). Indeed, several other studies have now reported a progressive exacerbation of pain with decreasing serum vitamin D levels and conversely, by increasing serum vitamin D levels through appropriate vitamin D supplementation, especially in vitamin D deficient patients, leads to an improvement in pain-relief (see Table 1). However, the potential underlying mechanisms by which vitamin D might exert its analgesic effects are poorly understood. In this review, we discuss pathways involved in pain sensing and processing primarily at the level of DRG neurons and the potential interplay between vitamin D/VDR and known specific pain signaling pathways including nerve growth factor (NGF), glial-derived neurotrophic factor (GDNF), epidermal growth factor receptor (EGFR), and opioid receptors. We also discuss how vitamin D might influence immune cells and pain sensitization as well as include a section on the increasingly important topic of vitamin D toxicity.

**Table 1:** Examples of clinical studies showing benefit in pain-relief following vitamin D supplementation.

		**Descriptors for population**					**Vitamin D treatment if any**				
**Type of pain**	**Vitamin D status**	**Age (year)**	**Geographic location**	**GenderM/F**	**Study size *(n)***	**Type**	**Duration**	**Dose**	**Route**	**Main findings/conclusion**	**References**
Back pain in overweight/obese	Deficient	31.8 ± 8.9	Australia	31M, 18F	54	Cholecalciferol (D3)	16 weeks	100,000 IU—Bolus followed by 4,000 IU per day for 16 weeks	Oral	No change in backpain intensity was noted; however, in markedly vitamin D deficient subjects (<30 nmol/L), back pain disability score was significantly improved	(7)
Diabetic neuropathic pain	Insufficient	43–78	Turkey	34F, 23M	57	Cholecalciferol (D3)	12 weeks	300,000 IU	im	Improvement in pain associated with small diameter c-fiber neurons (electric shock pain and burning pain) was reported after a single dose of IM vitamin D3	(8)
Menstrual pain in dysmenorrhea patients	Insufficient; deficient; severely deficient	18–30	Turkey	F 100	100	Cholecalciferol (D3)	3 months	Up to 1,040 IU (insufficient); up to 1,950 IU (deficient); up to 2,990 IU (severely deficient) for 2 months. In the third month all patients received 780 IU	Oral	The vitamin-D replacement therapy led to a significant decrease in pain symptoms based on pain visual analog scale. Benefits was greatest in severely vitamin D deficient patients	(9)
Low back pain	Insufficient; deficient	44	India	M37, F31	68	Cholecalciferol (D3)	8 weeks	60,000 IU/weekFor those patient with vitamin D <5 ng/ml received 60,000 IU/day for 5 days and then 60,000 IU/week for the next 8 weeks. Treatment was stopped for patients who achieved vitamin D level >60 ng/ml	Oral	Significant reduction in pain (VAS) and improvement in functional ability was observed at 2, 3, and 6 months. Interestingly, progressive improvement from 2, 3 to 6 months are both VAS and functional ability	(10)
Cancer	Insufficient; deficient	62.4 ± 13	Sweden	36M, 42F	78	Cholecalciferol (D3) dissolved in Miglyol	3 months	4,000 IE/day	Oral	Improvements in pain management (reduced Fentanyl dose) was seen as early as 1 month after treatment. After 3 months, significantly reduced antibiotic usage	(11)
Osteoarthritis	Insufficient; deficient	64.58 ± 0.55	Thailand	17M, 158F	175	Ergocalciferol (D2)	6 months	40,000 IU/week	Oral	Ergocalciferol supplementation decreased pain (VAS), improved LDL cholesterol levels, reduced oxidative protein damage and improved quality of life in osteoarthritis patients	(12)
Growing pains	Insufficient; deficient	10	Italy	18M, 15F	33	Cholecalciferol (D3)	3 months	40,000–100,000 IU/week	Oral	After the first 3 months of vitamin D treatment significant >60% reduction in pain intensity was reported	(13)
Locomotion and daily activities in elderly	Insufficient	58–89	Romania	17M, 28W	45	Cholecalciferol (D3)	12 months	125 μg	Oral (fortified in bread)	Improvement in reported pain symptoms	(14)
Sickle cell disease	Insufficient; deficient	13.2 ± 3.1	USA	19M, 27F	39	Cholecalciferol (D3)	6 weeks	40,000–100,000 IU/week	Oral	Significant reduction in the number of pain-days per week was reported at week 8 following treatment with cholecaciferol supplementation	(15)
Chronic pain	Insufficient; deficient	44.5	USA	M 28	28	Cholecalciferol (D3) [insufficient group]; Ergocalciferol (D2) [deficient group];	3 months	D3 1,200 IU/day; D2 50,000 IU/week	Oral	Reduced pain and reduced number of pains was reported following vitamin D supplementation in patients with multiple areas of chronic pain	(16)

## Vitamin D Absorption, Biosynthesis and Tissue Distribution

Vitamin D, although identified as a fat-soluble vitamin, is increasingly being recognized as a prohormone. Traditionally, vitamin D was considered to be essential for calcium and phosphate homeostasis and thereby, bone health, and its deficiency causes rickets and osteomalacia. Recent evidence, however, advocates a role for vitamin D that extends beyond bone metabolism (6). Our body obtains vitamin D both directly from the diet, albeit from a narrow range of food sources, and through biosynthesis in the skin. Vitamin D3 is the natural form of vitamin D and is produced from 7-dehydrocholesterol in the skin. UVB irradiation helps convert 7-dehydrocholesterol, the precursor in the skin, into pre-vitamin D3 and then to vitamin D3. Synthesis in skin serves as an essential source of vitamin D3 and depends crucially on season and geographical latitude (17). Studies involving human and pig models indicate that vitamin D3 is predominantly distributed in fat tissue (about three quarters) and in smaller amounts in muscles, liver, and skin. Distribution of 25-(OH)D3 was somewhat similar within compartments (serum, 30%; muscle, 20%; fat, 35% and 15% in other tissues) (18). The body fat content has also been reported to inversely correlate with serum levels of 25-(OH)D3, which could indicate that those with high body fat content (obese populations) might be at risk of vitamin D3 deficiency (19, 20).

## Vitamin D Metabolism

Vitamin D3, by itself, is not biologically active. 1,25-dihydroxy vitamin D3 (1,25-(OH)2D3) is the biologically active form of vitamin D and is produced by hydroxylation of vitamin D3 in two distinct steps and locations. Once synthesized (or absorbed from diet), vitamin D and its metabolites are bound to vitamin D-binding proteins (DBPs), which allow their transport in the blood. DBPs transport vitamin D3 to the liver where the first hydroxylation reaction occurs at C-25 position to produce 25-(OH)D3. 25-(OH)D3 is the major circulating form of vitamin D and hence is used as a biomarker of vitamin D status in the body. CYP2R1 is the key 25-hydroxylase of vitamin D in the liver (21). 25-(OH)D3 is then transported to the kidney where it is filtered and taken up into proximal renal tubule epithelial cells by the action of cell surface receptors for DBP, and megalin/cubulin complex (22, 23). In proximal renal tubules, 25-(OH)D3 is hydroxylated at the C-1 position by renal 25-(OH)D3 1α hydroxylase (CYP27B1), to produce 1,25-(OH)2D3, which is also known as calcitriol and is biologically active (24, 25). The circulating levels of 1,25-(OH)2D3 is regulated by the action of CYP24A1, another hydroxylase of C-24, to generate 24,25-(OH)2D3, which can allow its excretion or reduce the pool of 1,25-(OH)2D3 available for C-1 hydroxylation (26). CYP24A1 also catalyzes conversion of 1,25-(OH)2D3 and 25-(OH)D3 into 1,25-(OH)2D3-26,23 lactone and 25-(OH)D3-26,23 lactone, respectively. Presence of CYP24A1 in most cells expressing vitamin D receptors (VDR), suggests that CYP24A1 can regulate not only the circulating concentrations of 1,25-(OH)2D3 but also modulate its cellular levels locally (27).

## Vitamin D Receptor (VDR) IS a Nuclear Transcription Factor

Vitamin D mediates gene expression through interaction with its receptor, VDR. The VDR has been shown to recognize the sequence for vitamin D binding response element (VDRE) in the DNA and also forms a heterodimer with retinoid X receptor molecule. The effect of VDR is dependent on several other, as yet unknown, factors including enzymatic activities such as, demethyltransferase and RNA polymerase that mediate the promotion or inhibition of gene expression. Interestingly some of these target genes include those that bind 1,25(OH)2D3 to activate it or that mark it for its degradation, suggesting a self-regulating mechanism (28) and perhaps restricting the possibility for vitamin D toxicity due to over-exposure to sunlight or dietary intake. Other implicated target genes are those encoding for proteins involved in pain signaling pathways, such as growth factor receptors, ion channels, neurotrophic factors, and proteins involved in modulating neuronal axon growth, thereby implicating that vitamin D has a role in pain sensing and processing (see below).

## Does Vitamin D PLay a Role in Sensing Pain (Nociception)?

Pain is a subjective sensory experience that involves multiple signaling pathways (29–31). However, there are several categories of pain. In the proceeding sections we will discuss the interplay between vitamin D and different types of pain.

## Acute Pain

The ability to feel acute “normal pain,” protecting the injured tissue of the body to facilitate recovery and the amelioration of the resulting pain from an injury, is one of mother nature's gift to mankind and the rest of animal kingdom. We evolved to feel pain because the sensation and pain-induced responses serve as an alarm system that is necessary for self-preservation, and this evolutionary advantage is maintained in every animal with a sensory nervous system.

## Chronic Pain

However, the “normal pain” can change to “abnormal pain” and thereby the benefit as a necessary alarm to self-preservation becomes instead incapacitating to the individual and is then seen as nature's curse. This change is often due to damage to the nervous system, resulting in a lowering of the pain threshold to otherwise innocuous stimuli such as warmth and gentle touch and is now recognized as painful (allodynia). Or, when the degree of pain from a painful stimulus becomes intensified/exaggerated (hyperalgesia). These “abnormal pain” conditions often fail to resolve within 3 months (chronic pain), long after the acute injury, and are generally intractable to conventional analgesia and become debilitating. Chronic pain is broadly defined as “pain caused by a lesion or disease of the somatosensory nervous system” (www.iaps.org). The lesion could be due to damage to the nervous system (as observed in neuropathic pain) or inflammation (as found in arthritis). There are several types of chronic pain- [chronic primary pain, chronic postsurgical and posttraumatic pain, chronic neuropathic pain, chronic headache and orofacial pain, chronic visceral pain, and finally chronic musculoskeletal pain (32)] of which lower back pain probably being one of the most frequently experienced chronic pain conditions, and has a clear association between deficiency in vitamin D levels and pain as a result of softening of the bone (6).

## Visceral Pain, Vitamin D and Gut Microbiota

**Figure 1:**
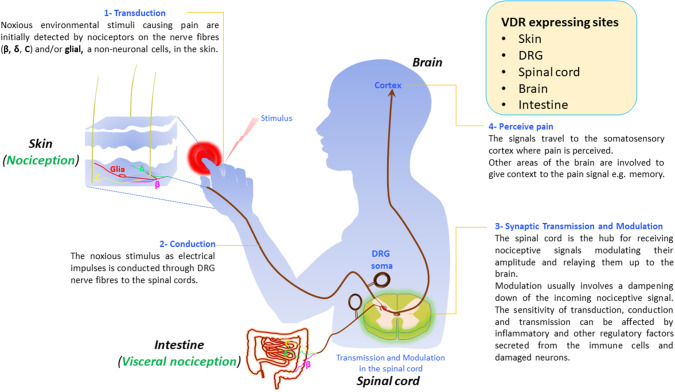
Vitamin D and its receptor in pain signaling pathways. Vitamin D is synthesized in the skin. Vitamin D receptor (VDR) is expressed in neurons in the skin, dorsal root ganglia (DRG), spinal cord, brain, and the intestine. Nociceptors, from the specific area of the periphery and viscera respond to environmental nociceptive stimuli such as thermal, mechanical, or chemicals by converting the stimuli into a chemoelectrical signal (1). The signal travels as a nerve impulse through dorsal root ganglia (DRG) nerve fibers (c, β, and δ) from the skin to the spinal cord (2) where it gets transmitted to secondary neurons in the dorsal horns of the spinal cord (3). After modulating the signal, the secondary neurons relay it to the cortex where the message gets decoded and pain is perceived (4).

Similar to nociception where the stimuli are sensed by the skin (see section below on Pain Signaling), the lining of the gut is also an “inside out” interface to our external environment and is essential in visceral nociception. Visceral pain is defined as that which originates from the tissues of the internal organs in the body, and pathologically affects more than 20% of the world's population. There is some homology between visceral nociceptors and those in the skin. For example, similar to the skin, the DRG nerve fibers that innervate the serosa and the muscle layer in the intestine also synapse in the spinal cord (Figure 1). Most visceral nociceptors are highly sensitive to pro-inflammatory signals, ischemia, and to chemicals released from the ischemic tissues.

Thus, visceral nociceptors are exquisite sensors of local changes in blood flow (ischemia) and inflammation within an organ, both of which can induce chronic visceral pain. Visceral pain can also emanate from factors in the lumen of the gut, including the microbiota and products derived from them.

The luminal microbiota aids digestion and generates short-chain fatty acids (SCFAs) that are an important energy source for the host. In addition, some of these SCFAs also serve as signaling molecules in host immunity and pain signaling. For example, we have shown, in mice, that SCFA can induce the release of glucagon-like peptide-1 (GLP-1) from intestinal L-cells (33). GLP-1 is a pleiotropic hormone mainly known for stimulating insulin release and appetite suppression. However, recent studies show that the GLP-1 receptor is also expressed in the sensory nerve fibers (34) and GLP-1 agonists have an anti-inflammatory action and can ameliorate pain (35, 36).

Several gastrointestinal neurotransmitters, in addition to their physiological role, also impact on pain sensing mechanisms. For example, the neurotransmitter serotonin is involved in motility of the gut, to propel the content in the lumen. However, it also modulates pain-sensing through potentiating the transient receptor potential cation channel subfamily V member 1 (TRPV1) channel (37, 38). Interestingly, the synthesis of serotonin in the enterochromaffin cell in the intestine is regulated in response to metabolites generated from gut microbiota (39). The importance of the gut microbiota in regulating gut motility was recently highlighted by Obata et al. (40). In this animal study it was shown that microbial colonization of the gut led to upregulation of several genes in the enteric neurons of the colon, including aryl hydrocarbon receptor, a transcription factor that regulates intestinal motility through neuronal programming (40). Furthermore, neuronal GPCRs that are involved in nociception are activated by SCFAs and other metabolites secreted or degraded from the gut microbiome (41, 42).

Under pathological conditions such as in irritable bowel syndrome, or in chemotherapy drug-induced mechanical hyperalgesia, a heightened pro-inflammatory state of the intestines is observed and results in exaggerated visceral pain. Thus, it is likely that the gut microbiota is an important regulator for the expression of pain-genes not only locally but also in distal organs, such as expression of Toll-like receptors (TLRs) and cytokines in the spinal cord that mediate visceral pain response, and are vital to the development of mechanical hyperalgesia (43, 44).

It is, therefore, perhaps unsurprising that dysbiosis of the gut microbiome results in chronic intestinal inflammation and other diseases. For example, the pathogenic bacteria- *Salmonella enterica*- commonly known to cause food poisoning, can disable the host's defenses against inflammation through activation of the TLR4. It also degrades intestinal alkaline phosphatase, an enzyme that is important in detoxifying endotoxin (lipopolysaccharide-phosphate) and thereby inducing gut microbiota-mediated autoimmunity and chronic inflammation (45).

Several studies indicate that vitamin D supplementation and/or deficiency changes the gut microbiota profile (46, 47); as such, this can potentially modulate visceral pain. Emerging data suggest vitamin D/VDR play a role in pain-sensing through modulating key pain-genes. Some of these pain-genes are common to both nociception and visceral nociception, for example, TRPV1, Toll-like receptor, trophic factors, such as NGF, BDNF and GPCR. The interplay between vitamin D and these pain-genes are further discussed below.

## Sensitization- Interplay Between Immune and Neuronal Cells

Spinal sensitization involves interactions between several neuronal and glial cells. The glial cells in the spinal cord play a significant role by responding to various cytokines and neurochemicals released from infiltrating macrophages, neutrophils and from the damaged peripheral nerve fibers. This response can be considered as part of protective measures, akin to acute pain, and vitamin D is thought to have a role in regulating the synthesis of cytokines (6).

There is growing evidence indicating that a family of TLR play a role in activating glial cells in the spinal cord and DRG neurons and thereby influence the sensitization process following nerve injury (48, 49). In a mouse neuropathic pain model of TLR2 knockout mice, the activity of the immune cells following the peripheral nerve injury is attenuated. Furthermore, reduced sensitivity to pain was observed in a behavior assay, suggesting that TLR2 could be a factor in neuropathic pain and in the activation of the glia in the spinal cord (50, 51). A similar result was also observed in TLR4 knockout mice (52). Vitamin D is known to be anti-inflammatory and inhibits the release of several cytokines and TLRs (53, 54). Expression of both receptors, TLR2 and TLR4, was shown to be modulated by vitamin D in human studies (55, 56). Identifying the molecular and neurochemical basis that cause/modulate the sensitization process may help enable us to identify therapeutic targets for the treatment of pain. In the next sections, we will focus on the potential ways by which vitamin D might play a role in the pain system.

## Pain Signaling

Skin is unique in that, apart from it being the most substantial organ system that protects the body from damage, it is also the largest damage-sensing organ and the only site for vitamin D synthesis in the body. Nociceptors, the specialized mammalian sensory neurons that react to tissue injury are critical components of the somatosensory nervous system, pain-sensing machinery, proposed initially by Charles Sherrington a 100 years ago (57). These neurons with their nuclei in the dorsal root ganglia (DRG) and their fibers stretching into the skin, the gut, and other organs in the body convey the information about potential harm to the central nervous system. However, a more recent study indicates that the ability to initiate the signal for sensing damage to the tissue is no longer exclusive to nociceptive free nerve endings but can also occur through glial cell signaling (58). Nonetheless, the role of nociceptors in pain signaling has been validated by several studies over the years, and their healthy development is a prerequisite for sensing pain.

Glial and Schwann cells, together with nociceptors, constitute the peripheral nervous system. These neuron and non-neuronal cell complexes require a healthy vascular system that supplies oxygenated blood and nutrients through small blood vessels. Vitamin D supplementation is known to significantly improve vascular functions in type 2 diabetic patients with vitamin D deficiency (59). Vitamin D supplementation was also shown to enhance myelination of DRG neurons and to regulate expression of genes involved in axon growth in an animal model of nerve damage (60). More recently, the existence of a previously unknown sensory organ: the skin glial cell that is intimately associated with unmyelinated small thin fibers which sense pain, has been reported. These nociceptive glio-neural complexes have been shown to evoke pain signaling, as evidenced by whole-cell current recordings in response to mechanical pressure (58) and therefore are essential to our ability to sense damage to the skin. However, further validation of this ground-breaking finding in humans is required. Several studies have reported an association between vitamin D insufficiency with pain in general and or following injury (61–63); however, knowledge of the underpinning molecular mechanisms is limited.

## Vitamin D and VDR in Pain Signaling Pathways

There now exists several clinical studies (see Table 1) and some animal studies, mostly in rodents (see Table 2), that show that vitamin D deficiency leads to a worsening of pain whereas appropriate vitamin D supplementation leads to better outcomes relating to pain. However, there are no definitive studies with knockout mice lacking VDR or one of the key vitamin D metabolizing enzymes that have been performed which directly links vitamin D/VDR to pain pathways. This is most likely as a result of these knockout mice being infertile (68).

**Table 2:** Selective examples of *in vivo* studies with Vitamin D receptor (VDR) KO mice and those examining the effect of Vitamin D and/or VDR on pain using animal models.

							**Vitamin D treatment if any**				
**Vitamin D status**	**Pain model**	**Species**	**Strain**	**Age**	**Body weight (g)**	**Pain behavior assay**	**Duration**	**Dose**	**Route**	**Main findings/conclusion**	**References**
Normal	Neuropathic pain	Rat	Sprague Dawley	NA	250–350	Mechanical nociceptive threshold method, Thermal cold allodynia	4 weeks	1,000 IU/kg	Gavage/diet	Vitamin D supplementation significantly reduced pain symptoms in monoarthiritic animals and accelerated recover from nerve injury compared with those on normal diet	(64)
Normal	Neuropathic pain (sciatic nerve injury)	Rat	Sprague Dawley	NA	200–250	Heat hyperalgesia (radian heat plantar), Cold (acetone), mechanical allodynia (von Frey)	Daily for 3 weeks post surgery	Up to 1,000 IU/kg	ip	Chronic vitamin D administration attenuates the behavioral scores of neuropathic pain	(65)
Deficient	Musculoskeletal and deep muscle Pain	Rat	Sprague Dawley	48 days	NA	Von Frey, Randall selitto test, paw pressure	4 weeks	2.2 IU/gm	Oral (diet)	Rats on cholecalciferol fortified diet showed less muscle pain than those on vitamin D deficient diet	(66)
Deficient (VDR KO)	NA	Mice	C57BL6	NA^*^	NA^*^	NA^*^	NA^*^	NA^*^	NA^*^	A viable transgenic animals using CRISPR Cas-9 were generated but infertile	(67)
Deficient (VDR KO)	NA	Mice	C57BL6	NA^*^	NA^*^	NA^*^	NA^*^	NA^*^	NA^*^	Vitamin D important in calcium homeostatic, bone formation as well as fertility	(68)

*NA is information not available*;

NA

* * is not applicable*.

Nonetheless, the hypothesis that vitamin D perhaps influences pain-signaling pathways is biologically plausible because vitamin D and/or vitamin D receptor gene expression has been shown in relevant tissues such as skin (pain signal transduction), DRG neurons (conduction), spinal cord (transmission/modulation), and brain (pain perception) (see also Figure 1).

## VDR and Vitamin D Regulating Enzymes Expression in Tissues Involved in Pain Signaling Pathways

Vitamin D receptor expression has been reported in the peripheral and central neurons involved in pain-sensing and processing. Indeed, there appears to be a local cell or tissue-specific processing of vitamin D in the neuronal system. Expressions of the transcript for nuclear vitamin D receptor and/or the enzymes regulating the active form of vitamin D levels have been shown in DRG neuron bare nerve fiber endings in the skin and the soma (69–71), in the spinal cord neurons (71, 72), and the brain (71, 73–75). The level of VDR transcript in DRG neurons is higher when compared to the different regions in the brain in humans, and interestingly, this expression pattern is inversed in the mouse (71). The reason for this is unclear, but it is noteworthy as mouse models of pain are widely used in studying pain signaling. Vitamin D activity is determined by two enzymes, CYP27B1 (activates vitamin D in the kidney) and CYP24A1 (inactivates the active vitamin D) (70). These two enzymes, along with VDR, are also expressed in nociceptor neurons as well as in the brain.

## Vitamin D and VDR in Pain Signal Transduction

Transduction of the pain signal involves the activation of ion channels that are expressed in nociceptive neurons in the skin in response to external stimuli, such as thermal, mechanical, or chemical inputs (see also Figure 1). Channels that are represented in the dermal and epidermal layers of the skin include the TRPV1, TRPV2, TRPV3, TRPV4 (for detecting heat); TRPM8, TRPA1 (for detecting cold), and TRPV4, TRPC (for detecting mechanical pressure sensing). The activation of these channels in response to stimuli leads to a transient change in cellular Ca^2+^ and Na^+^ concentration in the nociceptive neurons and thereby affecting their intrinsic electrical properties. The sensitivity of the skin to pain, touch or temperature differs depending on the sensory nerve ending whose sensitivity determines the kind of sensation evoked from the spot. The external stimulus detected by these receptor channels is then converted into an electrical signal and is transmitted along the axon by voltage-gated sodium channels. Therefore, for example, we and others have shown that a mutation in sodium channel Nav1.7 results in a complete loss of pain sensation (29, 76) or in extreme pain phenotypes (77, 78). Vitamin D, which is synthesized in the skin is thought to interact with nociceptive neuron nerve endings in the skin to directly sense noxious proinflammatory stimuli (79), and also to control TRPV1 channel activity in T cells (38, 80).

Taken together, the above findings suggest that VDR may play a role in modulating the expression of pain-genes, for example, those involved in the development of neurons and Schwann cells (60, 81) as well as ion channels expressed in nociceptor neurons that innervate the skin as discussed above. The presence of a tissue-specific “vitamin D enzyme system” that regulates vitamin D activity locally in specific tissues or cells including neuronal cells implies that the active vitamin D levels might be different from those in the general circulation. Therefore, alterations in the expression or function of vitamin D regulating enzymes, VDR expression, VDR targets in the skin and/or in sensory neurons or the associated glial cells could likely impact on chronic pain disorders listed above including neuropathic pain (82) and painful diabetic neuropathy (83).

## The Interplay Between NGF and Vitamin D in Pain Signaling

Studies *in vivo* and *in vitro* have shown that vitamin D increases nerve growth factor (NGF) expression from the DRG neurons innervating the skin in rats (84), as well as in hippocampal neurons (85). NGF is a necessary neurotrophic factor for the development and maturity of the nociceptors. It is composed of three subunits, 2-α, 2-γ and 1-β, and primarily exists in its precursor proNGF form. Cells that produce NGF include macrophages, mast cells, and bone marrow-derived macrophages and keratinocytes. The levels of NGF in disease conditions seems to increase in response to inflammation (86, 87).

NGF's role in pain signaling has been clearly demonstrated in families with a hereditary mutation in NGF or its receptor, tropomyosin kinase receptor type A (TrkA) (which is a typical tyrosine kinase receptor), resulting in a pain-free phenotype and impairment in the sensing of temperature (88, 89). NGF also stimulates the release of calcitonin gene-related peptide (CGRP) from the DRG peripheral endings (90). CGRP is thought to promote and maintain sensitized nociceptive neurons which implicates its role in chronic pain. The sensitization is also enhanced by increasing the insertion of TRPV1, the heated gated ion channel into the cell surface membrane, which is facilitated by NGF (91).

Furthermore, the transcription level of various isoforms of sodium channels (e.g., Nav1.6, Nav1.7, Nav1.8, and Nav1.9) is modulated by NGF and ultimately results in an increase in sodium current density and the floodgates to nociception, primarily through Nav1.8. The development of hyperalgesia during inflammation is also thought to arise from an increase in Nav1.7 expression promoted by NGF (92, 93). Since Nav1.7 expression is restricted to DRG neurons only, selective drugs modulating its channel activity have the potential to be a useful therapy for chronic pain (29). In a more recent study, lack of NGF is reported to cause retraction of nociceptors from the skin (94), hence severely affecting pain signaling pathways.

These studies suggest that NGF is critical for nociceptor neuron development and in pain processing; however, it is not clear whether this is a direct effect of vitamin D on NGF or if the outcome is indirect via extranuclear or nuclear signaling pathways.

## Vitamin D in Regulating GDNF Signaling

The glial cell line-derived neurotrophic factor (GDNF) is yet another neurotrophic factor that is expressed in a small population of DRG neurons (95, 96) and is implicated in promoting the survival and activity of large cutaneous sensory, proprioceptive neurons (97). In an animal model of neuropathic pain (spinal nerve ligation), GDNF has been shown to reverse the sensory abnormality induced by nerve damage and thereby ameliorate the pain, possibly thorough a tetrodotoxin (TTX) sensitive channel inhibition (98, 99). The neuropathic pain, following nerve damage is thought to be due to ectopic activity in the damaged myelinated TTX-sensitive neurons, i.e., those nociceptor neurons expressing, for example, fast-acting and fast-inactivating Nav1.7 channels. The utility of this channel has been exemplified in conditional deletion of Nav1.7 in nociceptive neurons, animals becoming pain-free in response to painful mechanical and inflammatory stimuli (100). Subsequent work by Minett et al. (101) showed a loss of pain-sensation to a noxious thermal stimulus. A similar pain phenotype has been documented in humans with inherited loss of function mutations in Nav1.7 (76). These findings demonstrate a central role for GDNF in pain signaling. Interestingly, a recent study shows that GDNF and its receptor, C-Ret are directly regulated by vitamin D (102). It is tempting to speculate that perhaps both vitamin D and its receptor could play a role in sodium channel-mediated neuropathic pain through modulating GDNF expression; however, experimental verification is needed.

## EGFR Signaling in Pain Processing: Interplay With Vitamin D?

The epidermal growth factor receptor (EGFR) and its downstream effectors have been recently identified as novel signaling pathways involved in pain processing (103) and their expression is also known to be regulated by the vitamin D pathway (104).

**Figure 2:**
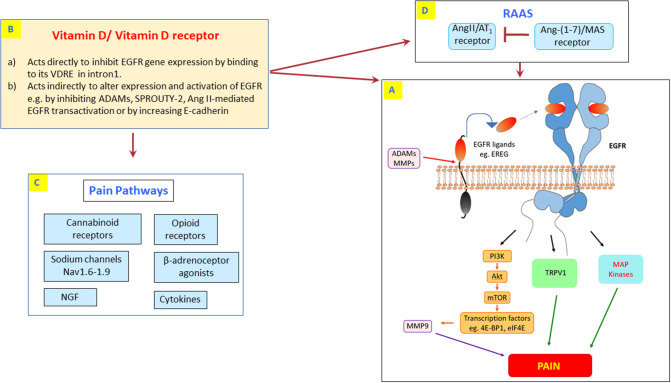
The potential cross-talk between vitamin D and EGFR pain signaling. **(A)** Highlights the known EREG/EGFR signaling pathways involved in pain sensing and processing including downstream PI3K/AKT, TRPV1, MAP kinases, and MMP-9 effector molecules. Vitamin D might exert its analgesic effect through direct inhibition of EGFR mRNA expression via binding to its VDRE in intron 1 **(B)**. Alternatively, vitamin D can act indirectly through modulation of other intermediary signaling cascades that lead to inhibition/modulation of EGFR **(B–D)**. This includes vitamin D-mediated regulation of known pain signals via opioid receptors, cannabinoid receptors, beta-adrenoceptors, sodium channels, cytokines, and NGF **(C)** as well as inhibiting the Ang II/AT1 receptor-mediated transactivation by the renin-angiotensin-aldosterone system (RAAS; **D**). In the RAAS, activation of the Ang-(1-7)/Mas receptor can counter-regulate the actions of Ang II/AT1 receptor and also inhibit EGFR signaling (113, 114) **(D)**. Additionally, vitamin D can also inhibit EGFR by inhibiting ADAMs, SPROUTY-2, or by increasing E-cadherin (**B**; see main text for details).

EGFR is a 175 KDa glycoprotein that is a member of the larger EGFR (also known as ErbB or HER) family of receptor tyrosine kinases which are important regulators of vital cellular processes such as cell growth, proliferation, differentiation, and apoptosis (105). The three other members of this family are known as EGFR2 (ErbB2, HER2), EGFR3 (ErbB3 or HER3), and EGFR 4 or (HER4) (106). EGFR signaling can be activated by either ligand-dependent or ligand-independent pathways such as via G-protein coupled receptor (GPCR) mediated transactivation that includes Angiotensin II (Ang II)-type 1 (AT1) receptors which are known to be involved in mediating several types of pain (107). In addition, EGFR transactivation has also been reported to occur via Beta-adrenergic receptors (108) as well as opioid and cannabinoid receptors (109–112) that also have important functions in regulating pain (see Figure 2).

For ligand-dependent EGFR activation, at least seven ligands have been described that bind to the mammalian receptor: Epidermal growth factor (EGF), transforming growth factor-alpha (TGF-α), heparin-binding EGF-like growth factor (HBEGF), betacellulin (BTC), amphiregulin (AREG), epiregulin (EREG), and epigen (EPGN). All EGFR ligands are synthesized as precursors that are tethered to the plasma membrane and require extracellular domain matrix metalloproteinases (MMPs) or members of the disintegrin and metalloprotease (ADAM) family (115). The soluble ligands released can then bind to and activate the EGFR (see also Figure 2). Autophosphorylation at specific tyrosine sites within the intracellular tyrosine kinase domain induces EGFR homo- or hetero-dimerization with other EGFR family members. This results in subsequent activation of downstream signaling pathways including MAP kinases and PI3K/AKT/mTOR (106), both of which are also known to be involved in pain regulation (116, 117).

EGFR is expressed widely in the cells of the body including epithelial cells, pain-transmitting neurons and keratinocytes of the skin—a primary source of vitamin D for the body (118, 119).

Dysregulated EGFR signaling is well-known in leading to several cancers but there is now strong evidence for its importance in other pathologies that result in pain as well as in the underlying mechanisms of pain sensing and processing. For example, in diabetes, microvascular dysfunction/microangiopathy is critically involved in the etiology of disease-associated neuropathy and subsequent neuropathic pain (120, 121). In this regard, we were the first to show that increased EGFR signaling was a critical mediator of diabetes-induced microvascular dysfunction (122–124). Mechanistic studies from our group identified that EGFR signaling via multiple pathways including those already known to be involved in pain signaling, such as MAP kinases and PI3K/AKT, were critical mediators of diabetes-induced microvascular dysfunction (125–128). Further we showed that EGFR inhibition could prevent vascular dysfunction by normalizing up to 90% of the 1,100+ gene changes observed during the development of diabetes-induced microvascular dysfunction and that upregulation of EGFR appears to be a key early change leading to vascular pathology associated with diabetes (125, 129, 130). Thus, our work implied that treatment of diabetes-induced vascular dysfunction- a precursor to the development of disease associated neuropathy- with EGFR inhibitors might also be potentially useful in preventing or at least delaying diabetic neuropathy and attenuating neuropathic pain. Vitamin D has been shown to prevent vascular dysfunction in diabetes patients and is known to attenuate the development of neuropathic pain (5, 59) suggesting that common pathways might be responsible in mediating vascular dysfunction and pain. Indeed, there appears to be a significant overlap of EGFR signaling pathways involved in microvascular dysfunction and the more recent studies on the role of EGFR and its ligands in pain processing including chronic neuropathic pain (118, 131, 132).

Martin et al. (103) reported that increased EGFR phosphorylation and upregulation of its ligand epiregulin (EREG) occurred in mouse models of chronic pain. In an elegant study conducted in human subjects, mice, and Drosophila, it was shown that activation of EGFR, specifically by EREG (and not other ligands such as EGF), activates DRG neurons and pain behaviors via mechanisms involving the TRPV1-a non-selective cation channel involved in nociception (discussed above), the PI3K/AKT/mTOR pathway as well as the matrix metalloproteinase, MMP-9 (103)-a molecule previously known to have a role in mediating inflammation and the early stages of neuropathic pain (133). In several mouse models of pain, intrathecal administration of EREG promoted nociception and led to pain hypersensitivity whereas treatment with preclinical (AG1478) or clinically used EGFR tyrosine kinase inhibitors (Gefitinib and Lapatinib) resulted in analgesia with efficacies and potencies comparable to that of morphine- an opioid analgesic. All EGFR inhibitors were effective in blocking inflammatory pain in two different animal models. They inhibited tonic inflammatory pain induced by formalin and completely attenuated thermal hypersensitivity induced by carrageenan- an inflammatory mediator. EGFR inhibitors were also completely and dose-dependently effective in reversing allodynia in three models of chronic pain including neuropathic pain. Knockdown of the EGFR ortholog specifically in peripheral neurons expressing pickpocket (ppk)—a nociceptive marker- in Drosophila resulted in prolonged withdrawal latencies in response to a heated probe at 46°C. This effect was reversed when EGFR gene was expressed in the neurons implying that the role of EGFR in pain processing is conserved across species. This study also showed that a single nucleotide polymorphism (rs1140475) in EGFR gene and one of its ligands (rs1563826 in epiregulin) was associated with development of chronic pain in humans suffering from temporomandibular disorder (103). Thus, this study demonstrated for the first time the direct association of EGFR and EREG with chronic pain in a clinical cohort of patients and further advocates the use of EREG/EGFR inhibitors as potential analgesics in the management of non-cancer pain in humans. These findings are supported by a recent study of lumbar radicular pain after disc herniation in rats, in which EREG was also thought to be released from herniated disc and mediated pain via PI3K/AKT pathway (134).

The analgesic effects of EGFR inhibition in several other human clinical studies have also been reported where diminished reporting of pain and an overall improvement in quality of life of patients was observed (131, 132, 135–137). In contrast to opioid analgesics, EGFR inhibitors showed no drug tolerance over the treatment period and the main side effect appeared to be skin rashes (131).

Taken together these studies suggest that EGFR is a key player in pain processing and sensation. Since it is already known that EGFR acts as an important hub and relay of signals from a variety of stimuli, its new role in processing pain signals would only provide added value to its proposed appellation as a “master hub or relay” of cell signaling (138) (see also Figure 2). Thus, inhibition of this important EGFR signaling hub by vitamin D might explain the latter's analgesic effects. Indeed, there are several studies suggesting that vitamin D inhibits EGFR gene expression either directly or indirectly via other intermediary signaling pathways (104, 107, 139–141).

Vitamin D through its nuclear receptor can directly regulate expression of EGFR mRNA by binding to VDRE identified in intron 1 of the EGFR gene (104). Interestingly, this study identified EGFR as the primary vitamin D/VDR target gene in these cells (104). Indirect actions of vitamin D in regulating EGFR signaling have also been reported. 1α,25(OH)_2_D_3_ increases the level of E-cadherin protein at the plasma membrane, which downregulates EGFR (142, 143), or by decreasing levels of SPROUTY-2, a cytosolic protein that normally inhibits EGFR ubiquitination, internalization and degradation (144, 145). A vitamin D3 derivative increased nerve growth factor levels in streptozotocin-diabetic rats (84) that is known to inhibit EGFR gene expression (146). Vitamin D supplements such as paricalcitol (a vitamin D analog) have also been reported to decrease EGFR signaling via inhibition of ADAM17 (141)-a key cleaving enzyme that mediates the release of EGFR ligands from the plasma membrane. In renal tubular cells, paricalcitol inhibited aldosterone-induced EGFR transactivation, and its resulting proinflammatory effects via inhibition of TGF-α/ADAM-17 gene expression (141). The fact that anti-inflammatory actions of paricalcitol in tubular cells were dependent on the inhibition of TGF-α/ADAM17/EGFR pathway may explain why both EGFR inhibitors and vitamin D are effective in models of inflammatory pain (64, 103). Vitamin D can also inhibit GPCR-mediated EGFR transactivation by blocking the actions of members of the renin-angiotensin system such as Ang II (107). The presence of these direct and indirect regulatory mechanisms of vitamin D in reducing EGFR signaling in pain neurons is not known but if reproduced in neuronal tissue, they might account for how vitamin D exerts its pain relief.

As a recent genetic study has implicated polymorphisms in EGFR and its ligand, epiregulin, to the development of chronic pain (103), it might be noteworthy that low 25(OH)D3 levels are associated with EGFR mutations in pulmonary adenocarcinoma (147). Whether hypovitaminosis D is responsible for EGFR polymorphisms in pain models is not known and is worthy of further study.

As EGFR is differentially expressed (129), it should also be noted that vitamin D regulation of EGFR signaling is also likely to be cell or tissue specific. In contrast to the above studies showing vitamin D-induced inhibition of EGFR in several cell types, in BT-20 breast cancer cells 1,25(OH)_2_D_3_ increases EGFR expression (148). Also, vitamin D supplementation with calcitriol led to the beneficial increase in neuregulin (a ligand for EGFR3 and 4 receptors) levels in the heart (149). Incidentally, Neuregulin is also required for remyelination and regeneration after nerve injury (150) and this may represent another mechanism by which vitamin D exerts its beneficial effects in models of neuropathic pain.

Like vitamin D, existing and newly discovered analgesic agents might also exert their pharmacological effects, at least in part, via EGFR inhibition. For example, opioids and cannabinoids are also known to regulate EGFR activity (109–112). Indeed, a novel role of cannabinoid receptor 2 in inhibiting EGF/EGFR pathway in breast cancer has recently been reported (111). Thus, the addition of vitamin D to the list of reagents modulating pain pathways via regulating EGFR signaling (see Figure 2) further implies that EGFR acts as a key convergent pathway in pain and as such might represent a novel pharmacological target for the development of new drugs and/or repurposing of existing anti-cancer EGFR inhibitors for pain therapy.

## Vitamin D and Regulation OF OPIOID Signaling

Despite the recent harrowing crisis of opioid dependency and its adverse sequalae in patients, opioids remain among the most widely used and indispensable analgesics for the treatment of moderate to severe pain. At present, no other oral therapeutic drug provides instant and effective relief of severe pain (151, 152). In rats, a vitamin D-deficiency diet was able to modulate the analgesic effect of the opioid, morphine- an effect that was blunted following administration of cholecalciferol (153). Cholecalciferol supplementation also induced dysregulation of opioid peptide genes, specifically POMC, PDYN, and PENK that code for endogenous opioids in the cerebrum (64, 154). In addition to modulating the expression of opioid agonists that are involved in pain perception, cholecalciferol supplementation did not affect the expression levels of opioid receptors. However, this supplementation was shown to dysregulate transcription levels of G protein subunits (Gαo) and effectors like subunits of the voltage-gated calcium channels (Cav2) and the adenylyl cyclase type 5 (AC5) enzymes as well as many other genes involved in opioid receptor signaling (64).

### Calcium Channels

Voltage-gated Cav2 channels are effectors of opioid receptors that initiate their activity through direct binding of G protein subunits. Following receptor activation, the Gβγ dimer released from Gαi/o protein binds to the intracellular loop between the transmembrane (TM) TM I and TM II of Cav2α1 channel subunits (155, 156). This binding shifts channel activation to more depolarized potentials (157) and decreases the activation kinetics of the calcium currents (158, 159), resulting in reduced synaptic vesicle fusion with the plasma membrane for neurotransmitter release (160). This opioid modulation of channel activity is thus involved in the analgesic effect of opioids.

Functional studies showed that expression of delta opioid receptors (DORs) in sensory neurons from rat trigeminal (161) or dorsal root ganglia (162) inhibited voltage-gated calcium channels following stimulation with DOR agonists. In addition, characterizing the different subtypes of calcium channels subject to DOR inhibition was elucidated using specific toxins and nominated Cav2.1 (P/Q-type) and Cav2.2 (N-type) channels as notable effectors of DORs (163–165) in DRG sensory neurons. Both Cav2.1 and Cav2.2 are localized in axon presynaptic terminals and play an important role in synaptic transmission between somatosensory neurons and dorsal horn neurons (166). However, while Cav2.1 is found in large-diameter DRG neurons, Cav2.2 is more expressed in small-diameter neurons (167–169). Surprisingly, similar to opioid receptors, vitamin D receptors have a widespread expression within the rat central nervous system and are found in high levels within distinct portions of the sensory, motor and limbic systems (72). Vitamin D receptors, like Cav2 channels, are also expressed in DRG neurons (69, 70, 170) and whether those receptors could affect opioid signaling by dimerizing with opioid receptors or by directly modulating the expression levels of Cav2 channels or other effectors, like the G protein-coupled inwardly rectifying potassium (GIRK or Kir3) channels involved in the analgesic effects of opioids (171–174), is still unknown.

### Adenylate Cyclase

The cAMP signaling pathway regulated via the stimulation of AC enzymes is one of the key mediators of opioid actions (172, 175, 176). Membrane-bound AC isoforms are highly expressed in the central nervous system and have overlapping expression patterns with opioid receptors. In the striatum, stimulation of DORs or mu opioid receptors (MORs) which are found to be co-expressed with AC5 (177–179), inhibits cAMP production and causes locomotor activation (176, 180). Those responses were attenuated in mice lacking AC5 (AC5^−/−^), making this enzyme an important component of the signal transduction mechanisms induced by opioid receptors.

cAMP signaling mediated by DORs may also contribute to the analgesic effects of opioids. In AC5^−/−^ mice, the analgesic effect of acute opioid stimulation was reduced compared to wild-type mice (180). Moreover, those results were specific for the AC5 enzymes since acute pain responses did not differ in mice lacking either AC1, AC8 or both isoforms compared to wild-type animals (181). However, like DOR and AC5, the vitamin D receptor is also expressed in the striatum and the prefrontal cortex (72). In addition, cholecalciferol supplementation was shown to induce down-regulation of AC5 gene in the cerebrum (64) and might than define a potential effect of vitamin D on the analgesic effects of opioids via the cAMP pathway.

## Vitamin D Levels and OPIOID Use

Low serum vitamin D levels have been linked to the development of opioid side effects and dependency. Among opioid-dependent patients recruited from the methadone maintenance treatment (MMT), more than half recorded a low vitamin D status (182). This common association deserves attention since high prevalence of chronic pain has also been observed in drug-dependent populations (183, 184). Chronic muscle and joint pain are common features of opioid withdrawal and are usually attributed to the effect of methadone (185). But, whether these conditions or other forms of chronic pain are affected by altered nutritional or metabolic factors related to vitamin D deficiency in drug-dependent populations is still unknown. Also, it should be noted that long-term therapy with some medications commonly used to treat chronic pain, such as steroids, antiepileptic drugs, and anticonvulsants can decrease levels of vitamin D (186–188). Therefore, it could be appropriate to recommend vitamin D supplements when levels are either deficient or insufficient with regards to the patient's medical conditions, current medications and skin exposure to sunlight. Nonetheless, vitamin D supplementation was recently shown to improve cognitive function and mental health in patients undergoing the MMT program (189).

Moreover, it has been suggested, using animal models, that ultraviolet B (UVB) light exposure induces strong analgesic effects due to elevation of endogenous opioids in the skin (190). This elevation in the POMC-derived peptide, β-endorphin, generated in response to UV radiation (191–193) was suggested to produce an opioid receptor-mediated addiction that leads to an increase in the nociceptive thresholds with pain relieving actions. However, since UVB light is responsible for the formation of vitamin D (194), it would be of interest to know if vitamin D plays a role in releasing β-endorphins to develop such addiction and if signaling pathways involved in this addiction are similar to those involved in exogenous opioid addiction.

It is known that sustained activation of opioid receptors results in compensatory mechanisms that increase cAMP production (195), referred to as AC superactivation- one of the key mechanisms leading to the development of physical dependence (196–198) and tolerance to opioids (172, 199–202). However, since cholecalciferol supplementation was shown to dysregulate AC5 gene expression (64), it might also be believed to play a critical role in modulating the development of opioid dependence and tolerance by affecting the AC superactivation mechanism. This correlation was partially proved by demonstrating the involvement of vitamin D deficiency in quickening the development of tolerance to opioid analgesics (153). In this study, rats raised for 3–4 weeks on a vitamin D-deficient rachitogenic diet developed tolerance to morphine faster than control rats, an effect that was prevented by cholecalciferol treatment.

Association between vitamin D levels and opioid use has also been investigated in human subjects. In an observational study, palliative cancer patients with 25-OHD levels <50 nmol/L required higher opioid (fentanyl) dose for pain relief compared to those with 25-OHD levels above 50 nmol/L (203). In a follow-up study, palliative cancer patients with 25-OHD levels <75 nmol/L were given vitamin D with a dose of 4,000 IU daily. After 1 month of supplementation, a decrease in fentanyl doses was observed compared to untreated controls (11). Together, these observations describe a significant correlation between low vitamin D levels and increased opioid consumption.

## Vitamin D Toxicity

Both hypovitaminosis D (low levels of vitamin D) and hypervitaminosis D (high Levels; also known as vitamin D toxicity) are linked with unwanted effects and symptoms. Low levels of serum 25(OH)D3 concentration typically below 12 ng ml^−1^ (30 nmol l^−1^) are associated with an increased risk of rickets/osteomalacia (204), however the precise serum levels of 25(OH)D3 that cause toxicity are not known. Those patients with serum levels >100 ng/ml are defined as being “at risk of toxicity” whereas serum values >150 ng/ml are considered “toxic” (205–207). However, another report suggests that serum levels above 50 ng/ml may be “at risk of toxicity” (208).

Hypervitaminosis D is usually diagnosed from presenting symptoms (see below), high serum 25(OH)D3 levels as well as elevated serum and urine level of calcium and reduced serum level of intact parathyroid hormone. Vitamin D toxicity is more likely in the modern age where excessive screening and media attention have led to widespread (mis)use of vitamin D supplements including as a result of over-prescribing by physicians (209, 210). It is thought to arise from chronic use of high dose vitamin D supplements and not from abnormally high exposure of skin to the sun or from eating a regular diet (211, 212). Data from National Health and Nutrition Examination Survey (NHANES) of US residents gathered between 1999 and 2014 found a 3% increase in the number of subjects taking potentially unsafe amounts of vitamin D—more than 4,000 international units (IU) per day- and a nearly 18% increase in the number of people taking more than 1,000 IU of vitamin D daily, well exceeding the Institute of Medicine recommended dose of 600 to 800 IU (213). These findings are consistent with another report showing concentrations of 25(OH)D3 have also modestly increased over this time frame from a similar population data-set (214). As a result of increasing use of vitamin D usage, a substantial increase in the number of reports of vitamin D intoxication, with the majority (75%) of reports published since 2010 (207). Hypervitaminosis D may also result from medication/prescription errors as well as formulation errors such as under reporting (wrong strength) on labels by unlicensed manufacturers of supplements and fortified foods (207, 215, 216).

Hypervitaminosis D, by definition, arises from elevated plasma concentrations of 25(OH)D3 and its metabolites such as 24,25(OH), 25,26(OH)2D3, and 25(OH)D3-26,23-lactone (217). Although the exact role of the individual metabolites is not well-understood, vitamin D toxicity is thought to arise from a saturation of its catabolizing enzymes such as CYP24A1 and hyperactivation of the vitamin D/VDR signaling pathways especially those regulating calcium levels (210, 218, 219).

It appears that short excursions into very high vitamin D serum levels such as after a single megadose of 300,000 IU might be safe (220) whereas chronic high doses of vitamin D in human subjects results in the following clinical manifestations: GI abnormalities >90%, Azotaemia ~80%, Polydipsia/Polyurea ~50% Encephalopathy 40%, Nephrocalcinosis 40%, Pancreatitis ~10% (207, 221, 222). Persistent vomiting as a result of vitamin D toxicity has also been recently reported (223). These clinical effects are largely thought to result from the vitamin-D-induced hypercalcemia that also leads to fatigue, generalized weakness and anorexia. The main mechanism of increased calcium in serum is due to vitamin D-induced bone resorption that can be blocked by bisphosphonates- known specific inhibitors of bone resorption- that are used clinically to treat hypercalcemia (224). In addition, hypervitaminosis D can also lead to increased absorption of dietary calcium from the GI tract, though this likely represents a minor contribution to the raised calcium levels in serum (224).

Hypervitaminosis D has been associated with increased risk of falls and bone fractures in post-menopausal women receiving high dose supplements (209). In extreme cases, vitamin D toxicity results in acute kidney injury (AKI). Chronic administration of mega doses (600,000 IU) has been shown to cause AKI and symptoms associated with hypercalcemia (210, 221, 225–227). In one study, 51 out of 62 patients receiving unusually high doses of vitamin D supplements developed AKI (226). The exact mechanisms for vitamin D associated renal complications are poorly understood but one rational explanation for the renal injury is hypercalcemia-induced severe dehydration. Renal toxicity may also be precipitated by and/or exacerbated by nephrocalcinosis (kidney stones) due to increased calcium deposition in renal tubules (228).

Vitamin D toxicity may also result from increased vitamin D/VDR signaling and/or decreased levels of catabolizing enzymes that could conceivably lead to hypervitaminosis D and subsequent toxic pharmacological effects (211, 212). However, as to what role is played by the “local vitamin D system” in cell/tissue specific toxicity remains poorly understood. For example, could overactivity of the “local vitamin D system” lead to a state of “local hypervitaminosis D” that then results in local cell/tissue toxicity? This would be important not only for its effects on the kidney but in other tissues such as in neurons involved in pain sensing and processing. Although, the contribution of the local vitamin D enzyme system to specific local tissue injury or damage is not well-understood, recent data suggests that the kidneys may be particularly prone to vitamin D toxicity. In addition to the normal uptake of vitamin D, the kidney proximal tubules also have the capability for re-uptake of DPB bound-vitamin D (DBP-D) through a Megalin and cubilin complex (229, 230). Thus, renal uptake of vitamin D by these multiple mechanisms could potentially result in higher accumulation and toxicity of vitamin D in the kidneys during hypervitaminosis D.

It should be noted, however, that vitamin D toxicity is rare and not all individuals with high serum 25(OH)D3 levels (even those with >150 ng/mL) manifest with clinical symptoms of vitamin D toxicity (24, 217, 218, 231, 232). Indeed, a recent study suggests that despite 167 patients having serum 25(OH)D3 levels >150 ng/mL, only 19 patients went on to develop vitamin D toxicity with AKI (210).

Vitamin D toxicity is more likely in extremes of age (children and elderly), in patients with impaired renal function, and those on certain prescription drugs such as thiazide diuretics (that decrease urinary calcium excretion) as their co-administration with vitamin D (that enhances intestinal calcium absorption) may theoretically result in hypercalcemia (233, 234).

Hypervitaminosis D and associated toxicity is largely preventable as it arises mainly from over-administration of high doses of vitamin D either by the patient, given by the physician/health care professional or because of manufacturer/formulation errors (207). Nonetheless, it is clear that further studies are needed to achieve a better understanding and definition of the precise cut-off levels for vitamin D toxicity in serum as well as in understanding the role of the local vitamin D system in mediating cell-specific toxicities (“local hypervitaminosis D”) that might arise even when circulating serum 25(OH)D3 levels are not abnormally high.

## Concluding Remarks and Perspectives

One in 5 of the adult population in the US is reported to suffer from chronic pain and 20% of these patients do not gain significant pain relief from currently available analgesic therapies. Vitamin D is speculated to provide clinical benefit in patients with chronic pain and several observational studies exist that have shown pain relief with vitamin D supplements (see Table 1). However, systematic reviews do not conclusively show patients benefit from vitamin D use in chronic pain (3, 235). There are multiple reasons for this discrepancy between the different clinical trials on vitamin D in pain which are discussed in more detail elsewhere [e.g., (3, 4)].

One of the concerns is the precise definition for serum 25(OH)D3 levels for its deficiency, for normal range and cut-off for toxicity. The difficulty in establishing pathophysiologically deficient 25(OH)D3 levels has been attributed to variations in methodological (statistical tools), the difference in experimental assays used (technical), and in the geographical latitude or other variations in the individuals being studied (236). Thus, it is argued that the so called “normal” range for serum 25(OH)D3 levels should be defined on an individual basis and in the clinical context (236). Serum variations can also arise from genetic polymorphisms in vitamin D processing enzymes and changes in vitamin D pharmacokinetics and pharmacodynamics. Another layer of complexity can arise from the specific variations in the individuals' disease state and this is particularly important in chronic pain that exhibits extreme heterogeneity amongst individuals and its perception can be very personalized. This leads to great challenges in the accurate assessment of pain especially when relying on self-reporting of pain by sufferers. Thus, there is an urgent need for the development of reliable biological markers of pain that can be accurately used in assessing pain in clinical trials. There is therefore a need for well-controlled large randomized clinical trials that account for the many variations, including having reliable biomarkers of pain, to conclusively determine the analgesic benefit of vitamin D in chronic pain.

The underlying molecular mechanism by which vitamin D/VDR modulates pain are not fully understood. In this review we have highlighted the potential interaction of pain signaling genes/pathways that are regulated by vitamin D/VDR. The reviewed literature suggests that vitamin D and/or its receptor can play a key role in modulating pain. Known pain genes such as NGF, GDNF, and EGFR, and opioid signaling are important components in pain signaling. We present here literature evidence for how vitamin D/VDR signaling might cross-talk with and regulate these genes that are critical in pain signaling. Further experimental studies *in vitro* and *vivo* are necessary to study these potential interactions specifically in pain models. Such studies could highlight the potential usefulness of vitamin D either alone or in combination with existing analgesics to better treat chronic pain.

## Author Contributions

AH and SA conceived the idea for the review. AH, SA, KN, NT, and VS wrote and edited the final draft of the manuscript.

## Conflict of Interest

The authors declare that the research was conducted in the absence of any commercial or financial relationships that could be construed as a potential conflict of interest.

## Footnotes

^1^www.iasp.org (accessed March 20, 2020).
